# The Prognostic Value of Serum Apolipoprotein A-I Level and Neutrophil-to-Lymphocyte Ratio in Colorectal Cancer Liver Metastasis

**DOI:** 10.1155/2022/9149788

**Published:** 2022-09-27

**Authors:** Chongkai Fang, Yue Huang, Chuyao Chen, Duorui Nie, Jietao Lin, Zhiwei Xiao, Saimei Li, Silin Liu, Rui Luo, Hongtong Lin, Chong Zhong, Xuewu Huang, Cui Shao

**Affiliations:** ^1^First Clinical Medical College, Guangzhou University of Chinese Medicine, Guangzhou, 510403 Guangdong, China; ^2^Lingnan Medical Research Center of Guangzhou University of Chinese Medicine, Guangzhou, 510403 Guangdong, China; ^3^Department of Hepatobiliary Surgery, First Affiliated Hospital of Guangzhou University of Chinese Medicine, Guangzhou, 510405 Guangdong, China; ^4^Cancer Center, First Affiliated Hospital of Guangzhou University of Chinese Medicine, Guangzhou, 510405 Guangdong, China; ^5^Department of Endocrinology, First Affiliated Hospital of Guangzhou University of Chinese Medicine, Guangzhou, 510405 Guangdong, China; ^6^Guangdong Provincial Hospital of Traditional Chinese Medicine, Guangzhou, 510120 Guangdong, China; ^7^Postdoctoral Research Station, Guangzhou University of Chinese Medicine, Guangzhou, China

## Abstract

**Background:**

Colorectal cancer liver metastasis (CRLM) is a high degree of malignancy with rapid disease progression and has a poor prognosis. Both serum apolipoprotein A-I (ApoA-I) and neutrophil-to-lymphocyte ratio (NLR) play key roles in anti-inflammation and antitumor. This study is aimed at evaluating the implication of serum ApoA-I level in combination with NLR in the prognosis of CRLM.

**Methods:**

We retrospectively analyzed the serum ApoA-I level and NLR in 237 patients with CRLM. Cox regression analyses were used to identify the independent prognostic significance of these indicators. Kaplan-Meier method and Log-rank test were applied to compute overall survival (OS). Both the ApoA-I and NLR were divided into three levels, according to their medians. A risk-stratified prediction model was established to evaluate the prognosis of patients with CRLM. The ROC curve AUC values were applied to evaluate the capability of the model.

**Results:**

Higher levels of ApoA-I and lower NLR were strongly associated with prolonged OS (Log-rank test, *P* < 0.05). The patients were then grouped into three queues according to the ApoA-I level and NLR. There was a crucial diversity in the OS (*P* < 0.001) between the high-risk (ApoA − I ≤ 1.03 g/L and NLR > 3.24), medium-risk (ApoA − I > 1.03 g/L or NLR ≤ 3.24) and low-risk groups (ApoA − I > 1.03 g/L and NLR ≤ 3.24). The AUC value of the prediction model (AUC = 0.623, 95% CI: 0.557-0.639, *P* = 0.001) was higher than other individual indicators (including ApoA-I, NLR, cT classification, and cN classification). Additionally, the association of the prediction model and cTN classification (AUC = 0.715, 95% CI: 0.606-0.708, *P* < 0.001) was better than the model and cTN classification alone.

**Conclusion:**

The combination of ApoA-I level and NLR could be a prognostic indicator for CRLM.

## 1. Introduction

Colorectal cancer (CRC) is one of the most common cancers worldwide. More than 50% of global new CRC and cancer-related death cases are found in China, Europe, and North America [1]. The incidence of CRC and mortality has decreased as a result of practical cancer screening measures. With the continued development of developing nations, it is anticipated that by 2035, there will be 2-5 million new CRC patients [2, 3]. Patients in the early stages of CRC can be treated with surgery for extended survival time. However, patients with advanced or metastatic CRC have limited surgical options and a poor prognosis despite other conservative treatments. Advanced CRC can lead to multiple organ metastasis, with the liver being the most common site [4]. Based on the published studies, patients with colorectal cancer liver metastasis (CRLM) have a median survival time of merely 19.7 months [5]. It is essential to predict the prognosis of advanced CRC patients with liver metastases.

Apolipoprotein is a member of the serum protein family that promotes lipid transportation and has recently been found to be involved in cancer metabolism and immunity [6]. Cancer cells obtain their required energy from lipids [7]. Apolipoprotein A-I (ApoA-I) is the main protein component of plasma high-density lipoprotein cholesterol (HDL-C). Approximately, 75% of the ApoA-I protein are synthesized and degraded in the liver cells [8]. ApoA-I protein facilitates ATP-binding cassette transporter 1 (ABCA1) in the reverse cholesterol transport (RCT) to maintain normal bile metabolism [9]. For instance, ApoA-I was demonstrated to reduce various oxidized lipids and enzymes involved in inflammatory mediators such as COX-2 in colon or ovarian cancer [10, 11]. However, ApoA-I may play anti-inflammatory and antitumor effects through other receptors, without affecting inflammatory mechanisms directly [9]. Some studies have discussed the anti-inflammatory and antitumor properties of ApoA-I, neutrophils, and lymphocytes. For instance, it was discovered that neutrophils would adhere to tumor cells if they overexpressed CD11b and intercellular adhesion molecule 1, but that ApoA-I would remove these molecules [12]. ApoA-I can also inhibit inflammation in hypercholesterolemic mice by reducing cholesterol accumulation in lymphocytes [13].

Inflammatory response plays a significant part in cancer development and progression [14]. The neutrophil-to-lymphocyte ratio (NLR), a ratio of the neutrophil, and lymphocyte counts measured in peripheral blood reflects the inflammatory response in the human body. Numerous researchers have assessed the prognostic value of NLR in various cancers [15]. An elevated NLR is a poor prognostic indicator in various cancers, including colorectal cancer [16], hepatocellular carcinoma [17], among others [18]. To date, the combination of NLR and ApoA-I to predict the prognosis of tumors has not been reported. Based on previous research, we primarily presented and investigated the combination of ApoA-I and NLR on the prognosis of CRLM.

## 2. Materials and Methods

### 2.1. Study Design

**Figure 1:**
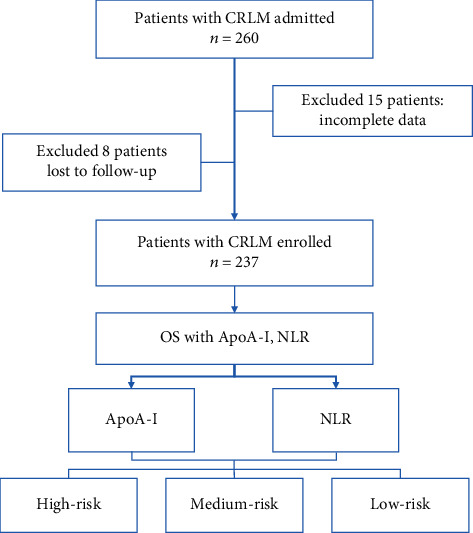
Study design and workflow.

The study gathered patients who met the diagnostic criteria for CRLM and hospitalized at the First Affiliated Hospital, Guangzhou University of Chinese Medicine, from January 1, 2008, to December 31, 2020. A total of 260 patients admitted for CRLM were screened, 23 of them were excluded due to incomplete data or lost to follow-up. Finally, 237 patients were included in this study (Figure 1). The inclusion criteria were as follows: over 18 years old; diagnosed with CRLM by medical imaging examination or postoperative pathological examination; no other evidence of distant metastasis; complete data on ApoA-I and NLR. Exclusion criteria for the study were as follows: other primary tumors or combined with other serious diseases; other diseases associated with blood lipid levels (such as diabetes, hyperlipidemia, or metabolic syndrome); receiving hormonal medication or taking any drugs that inhibit lipid metabolism; absence of follow-up records.

The study protocol was approved by the Ethics Committee of First Affiliated Hospital, Guangzhou University of Chinese Medicine. It was in line with the ethical principles established in the Declaration of Helsinki.

### 2.2. Methods for Measurement

Patients diagnosed with CRLM were screened using a blood test before receiving drug therapy for CRLM. For blood cell testing, including measuring levels of ApoA-I and NLR, 5 ml of fasting venous blood was taken from all patients and placed in K2-EDTA disposable anticoagulant vacuum tubes. After mixing, blood was collected for examination. ApoA-I was detected using a Cobas 8000 automatic biochemical analyzer (Roche). The NLR was tested using a BC-6900 automatic blood cell analyzer (Mindray, Shenzhen).

Through the hospital's electronic medical record system, the duration of follow-up was established to be from the first day of hospitalization to December 31, 2021. The variables (ApoA-I and NLR) were classified using the median value as the cut-off point. Thus, the cut-off level for ApoA-I was 1.03 g/L, and NLR was 3.24. We used the ApoA-I and NLR to analyze whether they were clinically associated with CRLM.

### 2.3. Statistical Analysis

Continuous variable data was represented by means ± standard deviations. Enumeration data were expressed as the number of cases (percentage). One-way variance analysis, Kruskal-Wallis *H* test, and the chi-square tests were used to evaluate statistical differences among groups. The Log-rank method was applied to test the differences in single-factor survival rates and to compare the distribution of survival curves. Survival curves were plotted using the Kaplan–Meier method. All significant variables for univariate Cox analysis were assessed using a multivariate Cox regression analysis to identify independent variables predicting survival in patients with CRLM. Exploratory subgroup analysis and interaction assays were used to investigate whether the association between ApoA-I and NLR and prognosis differed by clinical characteristics. The performance of the model was evaluated according to the ROC curve AUC value by the R *CoxBoost* package. Analyses were performed using the statistical software R (http://www.R-project.org) and Empower States (http://www.empowerstates.com). Descriptive and comparative statistical analyses were performed using SPSS 26.0. The figures were made using GraphPad Prism 9. All tests were two-sided, with a statistical significance level set at 0.05.

## 3. Results

### 3.1. Correlation of Serum ApoA-I Level, NLR, and Clinical Features

Of the 237 enrolled patients, 158 (66.7%) were male and 79 (33.3%) were female. The male-to-female ratio was 2 : 1, with more male patients than female patients. The age ranged from 24 to 83 years, with the median and mean age being 62 years and 60.48 years, respectively. The serum levels of ApoA-I and NLR are demonstrated in Table 1. Most CRLM patients with Karnofsky Performance Status Scale (KPS) scores ≥ 80 and patients with primary surgery had higher ApoA-I levels (*P* < 0.001, *P* = 0.009) and lower NLR (*P* = 0.026, *P* = 0.007). Patients with chemotherapy had significantly lower NLR levels than those in the control group (*P* < 0.001).

### 3.2. Patients' Characteristics and Kaplan–Meier Survival Analysis in CRLM patients

Univariate and multivariate Cox analyses were used for the assessment of the correlation between clinical features (including sex, age, ApoA-I levels, NLR, cT classification, cN classification, degree of tumor differentiation, number of liver metastases, type of liver metastases, pathological type, KPS scores, primary surgery, chemotherapy, targeted therapy, topical treatment, metastasis surgery, and tumor location) and overall survival (OS). Univariate Cox analysis, as shown in Table 2, demonstrated that ApoA-I levels (hazard ratio [HR]:0.655, *P* = 0.003), NLR (HR: 1.583, *P* = 0.001), cT classification (HR: 1.429, *P* = 0.012), cN classification (HR: 1.601, *P* < 0.001), the number of liver metastases (HR: 2.056, *P* < 0.001), type of liver metastases (HR: 0.539, *P* < 0.001), KPS scores (HR: 1.592, *P* = 0.005), tumor location (HR: 1.482, *P* = 0.012), chemotherapy (HR: 0.543, *P* < 0.001), topical treatment (HR: 0.684, *P* = 0.023), metastasis surgery (HR: 0.531, *P* = 0.002), and primary surgery (HR: 0.439, *P* < 0.001) were significantly associated with OS. Multivariate Cox analysis identified ApoA-I levels (HR: 0.717, 95% confidence interval [95% CI]: 0.527–0.976, *P* = 0.034), the number of liver metastases (HR: 1.760, 95% CI: 1.215-2.599, *P* = 0.004), type of liver metastases (HR: 0.598, 95% CI: 0.409-0.862, *P* = 0.007), tumor location (HR: 1.530, 95% CI: 1.086–2.128, *P* = 0.013), cN classification (HR: 1.422, 95% CI: 1.047–1.927, *P* = 0.024), primary surgery (HR: 0.659, 95% CI: 0.466-0.935, *P* = 0.019), and chemotherapy (HR: 0.628, 95% CI: 0.452-0.880, *P* = 0.006) as being significant independent predictors of survival in patients with CRLM.

**Figure 2:**
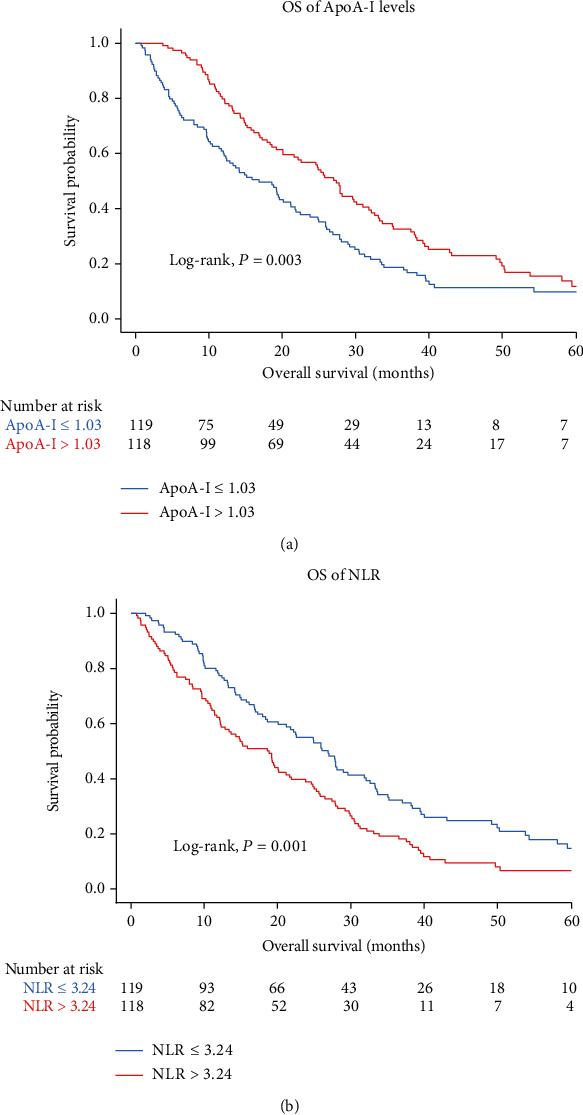
Kaplan-Meier curves of OS according to the ApoA-I (a) and NLR (b).

Kaplan–Meier curves showed that lower ApoA-I levels (*P* = 0.003) and higher NLR (*P* = 0.001) were significantly associated with a poorer OS among patients with CRLM (Figure 2). Thus, the analyses indicated that pretherapeutic ApoA-I level and NLR could affect the prognosis of patients with CRLM.

### 3.3. Stratified Analysis and Associations Between ApoA-I, NLR, and Prognosis by Clinical Features

**Figure 3:**
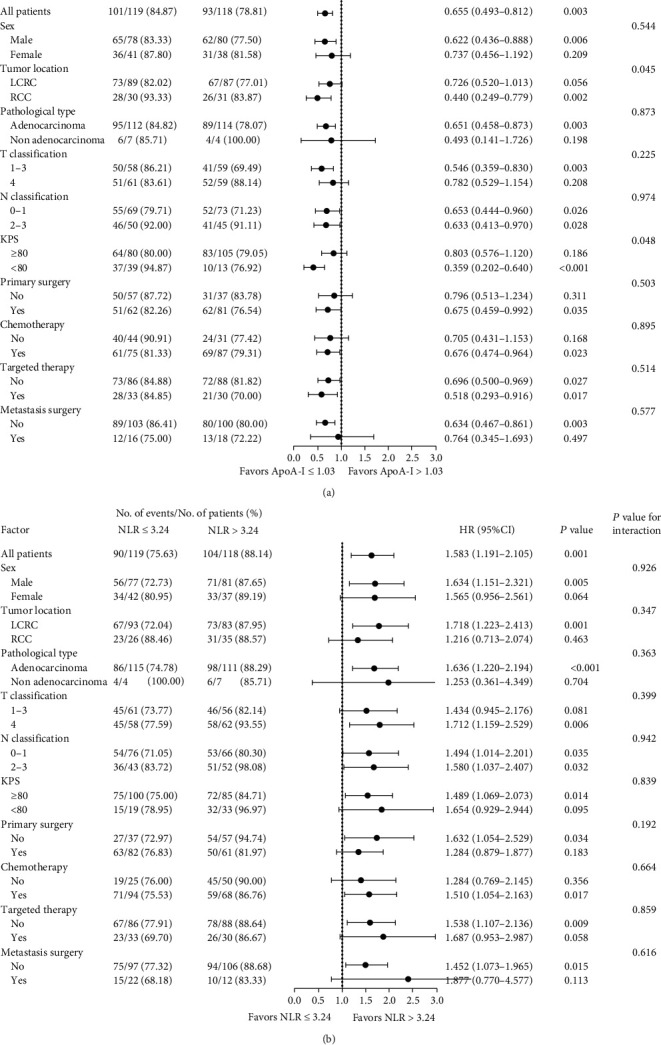
Stratified associations between ApoA-I (a), NLR (b), and prognosis by clinical features.

In light of the unbalanced baseline between the two groups (group classified according to the median value of ApoA-I or NLR), we assessed the interaction of ApoA-I levels and NLR with clinical features separately and performed an exploratory stratified analysis (Figure 3). We found that only ApoA-I level had a significant association with tumor location (*P* for interaction = 0.045) and KPS scores (*P* for interaction = 0.048) on prognosis. Stratifying the clinical characteristics further, the associations between ApoA-I and prognosis were more evident in the groups with RCC (*P* = 0.002) and KPS scores < 80 (*P* < 0.001) than in those with LCRC and KPS scores ≥ 80. The effect of ApoA-I and NLR on OS was consistent across the remaining subgroups.

### 3.4. Establishing a Novel Risk-Stratified Prognostic Model Using ApoA-I in Combination with NLR

**Figure 4:**
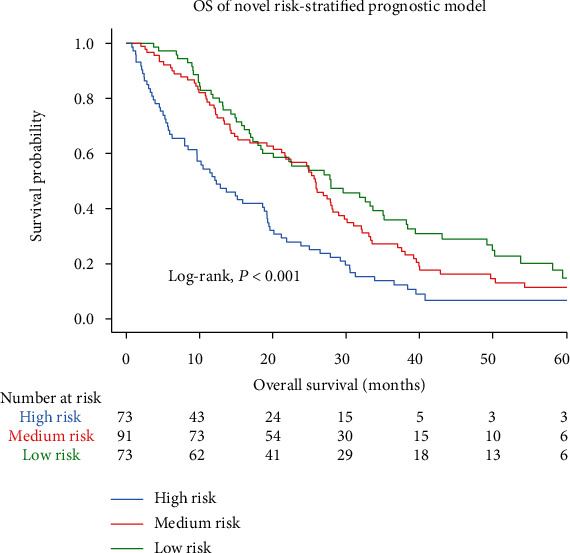
A novel risk-stratified prognostic model constructed by three categories.

Based on the ApoA-I level and NLR as independent risk factors for CRLM, we combined ApoA-I and NLR to construct a predictive model. Low levels of ApoA-I or high levels of NLR were associated with a poor prognosis of CRLM. To further explore the predictive value of the two indexes for poor prognosis, we stratified patients with CRLM into three categories: high, medium, and low risk. The categories are shown as follows: high-risk (ApoA − I ≤ 1.03 g/L and NLR > 3.24), medium-risk (ApoA − I > 1.03 g/L or NLR ≤ 3.24), and low-risk groups (ApoA − I > 1.03 g/L and NLR ≤ 3.24). The OS of the high-risk group of patients was poorer than that of the other two groups (Figure 4, Log-rank, *P* < 0.001).

### 3.5. Prognostic Values of The Individual ApoA-I level, NLR, and Their Combinations in CRLM Patients

**Figure 5:**
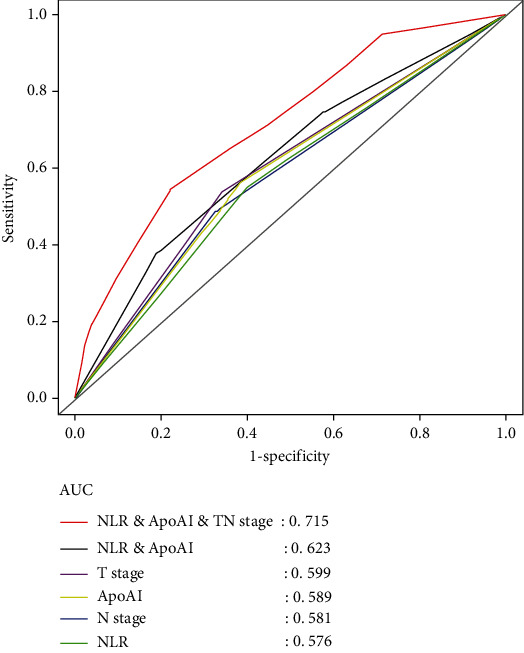
Comparison of the prognostic predictive ability of the risk-stratified prognostic model in CRLM patients.

**Figure 6:**
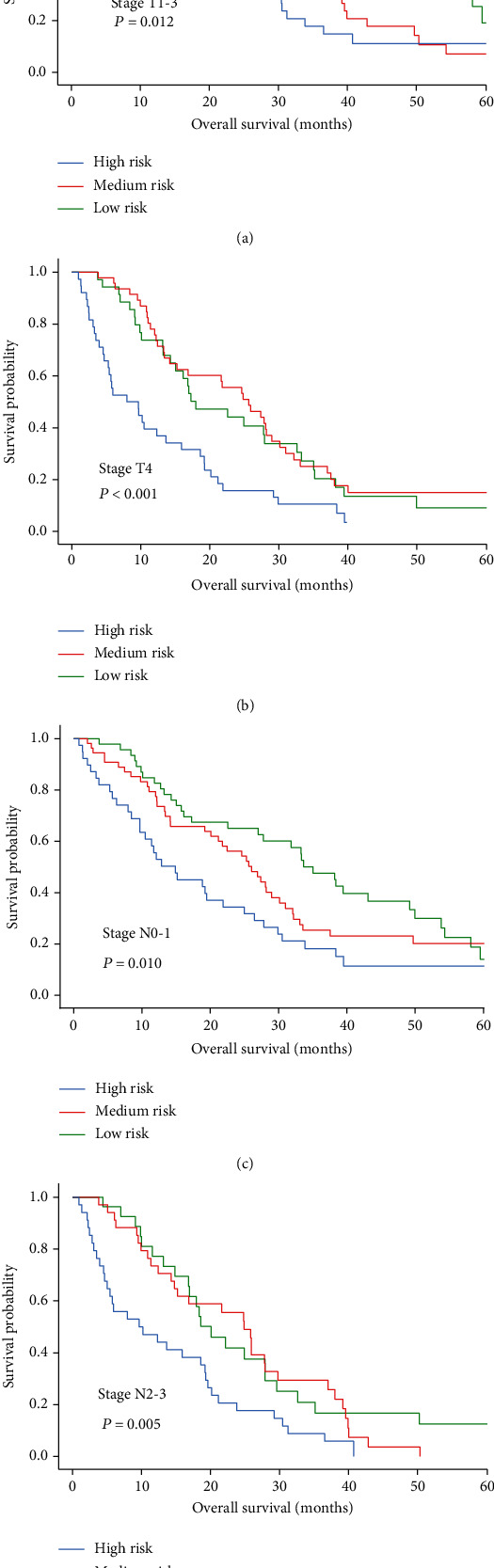
Prognostic significance of risk-stratified prognostic models in CRLM at different clinical stages by Kaplan-Meier survival curves. (a) OS in patients with CRLM in stages T1-3; (b) OS in patients in stage T4 CRLM; (c) OS in patients with CRLM in stages N0-1; (d) OS of patients with stages N2-3 CRLM.

We plotted ROC curves to assess the prognostic predictive ability of ApoA-I levels, NLR, cT classification, cN classification, ApoA-I combined with NLR, and combinations of the above in CRLM (Figure 5). Based on the area under curve (AUC), the prediction of the risk-stratified prognostic model was 0.623 (95% CI: 0.557–0.639, *P* = 0.001), which was higher than other individual indicators (ApoA-I: AUC = 0.589, 95% CI: 0.557–0.639, *P* = 0.003; NLR: AUC = 0.576, 95% CI: 0.539–0.625, *P* = 0.001; cT classification: AUC = 0.599, 95% CI: 0.527–0.614, *P* = 0.006; cN classification: AUC = 0.581, 95% CI: 0.514–0.602, *P* = 0.003). Furthermore, we combined the cTN classification and prognostic model for ROC analysis. The results showed that the combined AUC (AUC = 0.715, 95% CI: 0.606–0.708, *P* < 0.001) was superior to the cTN classification or prognostic model. Moreover, to examine the prognostic significance of the prognostic model in patients with CRLM, we analyzed the prognostic effects in subgroups stratified by cTN classification. OS was significantly worse in high-risk patients compared to those at low and medium risk (*P* = 0.012, *P* < 0.001, *P* = 0.010, *P* = 0.005 for T1-3, T4, N0-1, and N2-3 stages, respectively), as shown in Figure 6.

## 4. Discussion

In this study, we assessed the prognosis of patients with CRLM using serum ApoA-I level and NLR. Univariate Cox analysis showed that ApoA-I level, NLR, cT classification, cN classification, the number of liver metastases, type of liver metastases, KPS scores, tumor location, chemotherapy, topical treatment, metastasis surgery, and primary surgery were associated with OS. Further multivariate Cox analysis suggested that higher levels of ApoA-I, metachronous liver metastases, left-sided colon cancer, primary surgery, chemotherapy, lower levels of cN classification (N0-1), and lower number of liver metastases (≤2) were associated with better OS. Based on Kaplan–Meier survival analysis at independent prognostic levels, patients with CRLM having high ApoA-I levels or low NLR had a prolonged OS and those having lower levels of ApoA-I or higher NLR were associated with shorter OS. Consequently, we combined ApoA-I and NLR to construct a risk-stratified prognostic model. The predictive risk model revealed that ApoA-I levels and NLR could be used to evaluate the prognosis of patients with CRLM and monitor the efficacy of treatment. In addition, we used AUC values to rate the prognostic predictive capacity of the prognostic model in CRLM and found that the AUC value of the model (AUC = 0.623, 95% CI: 0.557–0.639, *P* = 0.001) was higher than those of ApoA-I and NLR alone. For the combination of the model and cTN classification, we also found that the AUC value of the model (AUC = 0.715. 95% CI: 0.606–0.708, *P* < 0.001) was superior to the cTN classification and prognostic model alone.

Moreover, we found a significant association of ApoA-I level, tumor location, and KPS scores on the risk of death according to the interaction assays. The relationship between ApoA-I level and prognosis was less apparent in those colon cancer in the left side and KPS scores ≥ 80 compared with those colon cancer on the right side and KPS scores < 80. The underlying causes of these differences must be investigated further.

Zhang et al. have shown that lipid metabolism is associated with tumorigenesis and tumor progression [19]. ApoA-I, the major protein component of high-density lipoprotein (HDL), is synthesized mainly in the liver and small intestine and is one of the member of the apolipoprotein A1/A4/E family [20].ApoA-I binds to ABCA1 on the cell membranes of hepatocytes and intestinal cells and mediates the production of new HDL particles. ApoA-I stabilizes ABCA1 on the hepatocyte and intestinal cell membrane to mediate phospholipids and free cholesterol flowing out to form new HDL particles. ABCA1 lipid efflux in peripheral tissues initiates RCT [21, 22]. In addition, ApoA-I leads to the maturation of HDL particles through activation of lecithin cholesterol acyltransferase [23]. In dish-shaped or more mature HDL particles, lipidified ApoA-I interacts with another transporter of the ABC family, ATP-binding cassette subfamily G member 1, further facilitating RCT [24]. ApoA-I is related to cholesterol transport, inflammatory, and immune response regulation. From the perspective of humoral arm of innate immunity, ApoA-I has proved to inhibit the formation of the complement terminal attack complex C5b-9 and contribute to complement clearance by interfering with C9 polymerization and incorporation membrane [25]. Among the antigen-induced mouse arthritis model, ApoA-I/HDL inhibited dendritic cell maturation and reactivity of Th1 and Th17 cells, resulting in alleviating arthritis [26]. ApoA-I levels are strongly related to therapeutic efficacy and the risk of various tumors. For example, by comparing the level of ApoA-I in patients with nasopharyngeal cancer and ovarian cancer before chemotherapy, a higher level of ApoA-I improved the overall survival after chemotherapy [27]. The Malmo Diet and Cancer Study showed that the incidence of colorectal cancer, lung cancer, and breast cancer is negatively correlated with HDL-C and ApoA-I levels [28]. A cross-sectional study in South Korea has also reported the correlation between HDL/ApoA-I levels and high risks of colorectal cancer, and in colonoscopy, the precancerous lesion of colorectal cancer (colon adenoma) is associated with a lower HDL level [29, 30]. Nonetheless, the predictive value of ApoA-I levels on OS in patients with CRLM remains unclear. Our analyses revealed that high ApoA-I level was significantly associated with prolonged OS. Li et al. discovered that elevated ApoA-I inhibits the movement and invasion of neutrophils at tumor sites rich in chronic inflammatory factors, thereby reducing the number of neutrophils infiltrating tumor tissue and ultimately increasing the survival rate [12]. Increasing ApoA-I levels by ApoA-I transgenic mice expression inhibited lymphocyte activation, decreased germinal center B cell numbers at length, and ameliorated glomerulonephritis [13]. These studies indicate that ApoA-I influences the number of leukocytes and the development of inflammatory responses in the body. Numerous researchers have revealed the prognostic value of NLR in various cancers. A study has shown that a high NLR was linked to shorter survival in laryngeal cancer patients treated with a preservation protocol [31]. Liu et al. found that NLR may be one of the prognostic indicators for the treatment of metastatic non-small cell lung cancer [32]. A study of thyroid cancer showed that preoperative NLR measurements were associated with pathological predictors such as tumor size [33]. There is an important link between high NLR and poor long-term survival rate and higher risk of colorectal cancer extrahepatic recurrence. Some studies support a high NLR predicts more aggressive systemic disease and may serve as a biomarker for preoperative risk stratification with patients undergoing CRLM resection [34].

In this retrospective study, NLR and ApoA-I levels were contrasted separately and combined to evaluate the prognosis of patients with CRLM. We discovered that ApoA-I combined with NLR could be used as a prognostic predictor for CRLM. However, there are some limitations to this study. Patients were sourced from a single center, and selection bias may exist. In addition, it would be worthwhile to investigate the prognostic capacities of our model in CRLM patients with coexisting diseases (such as diabetes, hyperlipidemia, or metabolic syndrome) that alter lipid levels. A prospective study with a more extensive and diverse patient population is needed to validate our findings.

Tumor patients need to undergo various examinations to evaluate treatment efficacy and the monetary cost is considerable. This study constructs a risk stratification model to assess the prognosis of patients with CRLM using serum ApoA-I level and NLR. In conclusion, this study provides a noninvasive and convenient indicator for long-term dynamic monitoring of patients.

## Figures and Tables

**Table 1:** The clinical characteristics of patients grouped according to ApoA-I level and NLR.

Variables	ApoA − I ≤ 1.03	ApoA − I > 1.03	*P* value^∗^	NLR ≤ 3.24	NLR > 3.24	*P* value^∗^
N	119	118		119	118	
Age	61.101 ± 12.321	59.847 ± 11.045	0.411	59.639 ± 11.600	61.322 ± 11.779	0.269
Sex			0.713			0.520
(i) Male	78 (65.546%)	80 (67.797%)		77 (64.706%)	81 (68.644%)	
(ii) Female	41 (34.454%)	38 (32.203%)		42 (35.294%)	37 (31.356%)	
Liver metastases number			0.359			0.155
(i) ≤2	29 (24.370%)	35 (29.661%)		37 (31.092%)	27 (22.881%)	
(ii) >2	90 (75.630%)	83 (70.339%)		82 (68.908%)	91 (77.119%)	
Type of liver metastases			0.430			0.189
(i) Simultaneous	92 (77.311%)	86 (72.881%)		85 (71.429%)	93 (78.814%)	
(ii) Metachronous	27 (22.689%)	32 (27.119%)		34 (28.571%)	25 (21.186%)	
Tumor location			0.852			0.169
(i) LCRC	89 (74.790%)	87 (73.729%)		93 (78.070%)	83 (71.681%)	
(ii) RCC	30 (25.210%)	31 (26.271%)		26 (21.930%)	35 (28.319%)	
Pathological type			0.362			0.347
(i) Adenocarcinoma	112 (94.118%)	114 (96.610%)		115 (96.639%)	111 (94.068%)	
(ii) Nonadenocarcinoma	7 (5.882%)	4 (3.390%)		4 (3.361%)	7 (5.932%)	
Degree of tumor differentiation			0.363			0.416
(i) Moderately	88 (73.950%)	94 (79.661%)		91 (76.470%)	91 (77.119%)	
(ii) Poorly	22 (18.487%)	14 (11.864%)		16 (13.445%)	20 (16.949%)	
(iii) Well	9 (7.563%)	10 (8.475%)		12 (10.084%)	7 (5.932%)	
cT classification			0.846			0.558
(i) 1-3	58 (48.739%)	59 (50.000%)		61 (51.261%)	56 (47.458%)	
(ii) 4	61 (51.261%)	59 (50.000%)		58 (48.739%)	62 (52.542%)	
cN classification			0.542			0.213
(i) 0-1	69 (57.983%)	73 (61.864%)		76 (63.866%)	66 (55.932%)	
(ii) 2-3	50 (42.017%)	45 (38.136%)		43 (36.134%)	52 (44.068%)	
KPS scores			<**0.001**			**0.026**
(i) ≥80	80 (67.227%)	105 (88.983%)		100 (84.034%)	85 (72.034%)	
(ii) <80	39 (32.773%)	13 (11.017%)		19 (15.966%)	33 (27.966%)	
Primary surgery			**0.009**			**0.007**
(i) No	57 (47.899%)	37 (31.356%)		37 (31.092%)	57 (48.305%)	
(ii) Yes	62 (52.101%)	81 (68.644%)		82 (68.908%)	61 (51.695%)	
Chemotherapy			0.076			<**0.001**
(i) No	44 (36.975%)	31 (26.271%)		25 (21.008%)	50 (42.373%)	
(ii) Yes	75 (63.025%)	87 (73.729%)		94 (78.992%)	68 (57.627%)	
Targeted therapy			0.688			0.688
(i) No	86 (72.269%)	88 (74.576%)		86 (72.269%)	88 (74.576%)	
(ii) Yes	33 (27.731%)	30 (25.424%)		33 (27.731%)	30 (25.424%)	
Topical treatment			0.421			0.098
(i) No	94 (78.992%)	88 (74.576%)		86 (72.269%)	96 (81.356%)	
(ii) Yes	25 (21.008%)	30 (25.424%)		33 (27.731%)	22 (18.644%)	
Metastasis surgery			0.691			0.068
(i) No	103 (86.555%)	100 (84.746%)		97 (81.513%)	106 (89.831%)	
(ii) Yes	16 (13.445%)	18 (15.254%)		22 (18.487%)	12 (10.169%)	

^∗^ *P* < 0.05 considered as statistically significant.

**Table 2:** Univariate and multivariate Cox analysis for OS in patients with CRLM.

Variables	Univariate analysis		Multivariate Analysis	
	HR (95%CI)	*P* value^∗^	HR (95%CI)	*P* value^∗^
Age	1.157 (0.861, 1.555)	0.321		
(i) <65 vs. ≥65				
Sex	1.305 (0.956, 1.781)	0.076		
(i) Male vs. Female				
Number of Liver metastases	2.056 (1.538, 2.749)	<**0.001**	1.760 (1.215, 2.599)	**0.004**
(i) ≤2 vs. >2				
Type of liver metastases	0.539 (0.401, 0.723)	<**0.001**	0.598 (0.409, 0.862)	**0.007**
(i) Simultaneous vs. Metachronous				
Tumor location	1.482 (1.047, 2.098)	**0.012**	1.530 (1.086, 2.128)	**0.013**
(i) LCRC vs. RCC				
Pathological type	0.816 (0.456, 1.460)	0.529		
(i) Adenocarcinoma vs. nonadenocarcinoma				
Degree of tumor differentiation	0.768 (0.506, 1.166)	0.092		
(i) Poorly vs. moderately vs. well				
cT classification	1.429 (1.077, 1.898)	**0.012**	1.277 (0.949, 1.720)	0.106
(i) 1-3 vs. 4				
cN classification	1.601 (1.189, 2.157)	<**0.001**	1.422 (1.047, 1.927)	**0.024**
(i) 0-1 vs. 2-3				
KPS scores	1.592 (1.092, 2.322)	**0.005**	1.396 (0.970, 1.977)	0.066
(i) ≥80 vs. <80				
Primary surgery	0.439 (0.316, 0.611)	<**0.001**	0.659 (0.466, 0.935)	**0.019**
(i) No vs. Yes				
Chemotherapy	0.543 (0.385, 0.767)	<**0.001**	0.628 (0.452, 0.880)	**0.006**
(i) No vs. Yes				
Targeted therapy	0.794 (0.584, 1.080)	0.161		
(i) No vs. Yes				
Topical treatment	0.684 (0.505, 0.926)	**0.023**	0.933 (0.648, 1.321)	0.700
(i) No vs. Yes				
Metastasis surgery	0.531 (0.378, 0.747)	**0.002**	0.978 (0.594, 1.560)	0.928
(i) No vs. Yes				
ApoA-I	0.655 (0.493, 0.872)	**0.003**	0.717 (0.527, 0.976)	**0.034**
(i) ≤1.03 vs. >1.03				
NLR	1.583 (1.191, 2.105)	**0.001**	1.159 (0.852, 1.578)	0.348
(i) ≤3.24 vs. >3.24				

^∗^ *P* < 0.05 considered as statistically significant.

## Data Availability

All data generated or analyzed in this study are included in this published article.

## Abbreviations

CRC: Colorectal cancer
ApoA-I: Apolipoprotein A-I
HDL-C: High-density lipoprotein cholesterol
HDL: High-density lipoprotein
ABCA1: ATP-binding cassette transporter 1
CRLM: Colorectal liver metastasis
RCT: Reverse cholesterol transport
NLR: Neutrophil to lymphocyte ratio
OS: Overall survival
HR: Hazard ratio
95% CI: 95% confidence interval

## Supplementary Materials

Supplementary Materials Normal values (ranges) of our laboratory.

## References

[1] Li N., Lu B., Luo C. (2021). Incidence, mortality, survival, risk factor and screening of colorectal cancer: a comparison among China, Europe, and northern America. *Cancer Letters*.

[2] Bray F., Ferlay J., Soerjomataram I., Siegel R. L., Torre L. A., Jemal A. (2018). Global cancer statistics 2018: GLOBOCAN estimates of incidence and mortality worldwide for 36 cancers in 185 countries. *CA: a Cancer Journal for Clinicians*.

[3] Arnold M., Sierra M. S., Laversanne M., Soerjomataram I., Jemal A., Bray F. (2017). Global patterns and trends in colorectal cancer incidence and mortality. *Gut*.

[4] Adam R., Vinet E. (2004). *Regional treatment of metastasis: surgery of colorectal liver metastases*. *Medical Oncology*.

[5] Chen C., Yi W., Zeng Z.-F. (2022). Serum apolipoprotein B to apolipoprotein A-I ratio is an independent predictor of liver metastasis from locally advanced rectal cancer in patients receiving neoadjuvant chemoradiotherapy plus surgery. *BMC Cancer*.

[6] Delk S. C., Chattopadhyay A., Escola-Gil J. C., Fogelman A. M., Reddy S. T. (2021). Apolipoprotein mimetics in cancer. *Seminars in Cancer Biology*.

[7] Vander Heiden M. G., Cantley L. C., Thompson C. B. (2009). Understanding the Warburg effect: the metabolic requirements of cell proliferation. *Science*.

[8] Georgila K., Gounis M., Havaki S., Gorgoulis V. G., Eliopoulos A. G. (2020). mTORC1-dependent protein synthesis and autophagy uncouple in the regulation of apolipoprotein A-I expression. *Metabolism*.

[9] Georgila K., Vyrla D., Drakos E. (2019). Apolipoprotein A-I (ApoA-I), immunity, inflammation and cancer. *Cancers*.

[10] Gao F., Chattopadhyay A., Navab M. (2012). Apolipoprotein A-I mimetic peptides inhibit expression and activity of hypoxia-inducible factor-1*α* in human ovarian cancer cell lines and a mouse ovarian cancer model. *The Journal of Pharmacology and Experimental Therapeutics*.

[11] Aguirre-Portolés C., Feliu J., Reglero G., Ramírez de Molina A. (2018). ABCA1 overexpression worsens colorectal cancer prognosis by facilitating tumour growth and caveolin-1-dependent invasiveness, and these effects can be ameliorated using the BET inhibitor apabetalone. *Molecular Oncology*.

[12] Li J., Wu Y. L., Li W. F., Ma J. (2021). Neutrophil to apolipoprotein A-I ratio as an independent indicator of locally advanced nasopharyngeal carcinoma. *Laryngoscope Investigative Otolaryngology*.

[13] Black L. L., Srivastava R., Schoeb T. R., Moore R. D., Barnes S., Kabarowski J. H. (2015). Cholesterol-independent suppression of lymphocyte activation, autoimmunity, and glomerulonephritis by apolipoprotein A-I in normocholesterolemic lupus-prone Mice. *The Journal of Immunology*.

[14] Grivennikov S. I., Greten F. R., Karin M. (2010). Immunity, inflammation, and cancer. *Cell*.

[15] Shaul M. E., Fridlender Z. G. (2019). Tumour-associated neutrophils in patients with cancer. *Nature Reviews. Clinical Oncology*.

[16] Grenader T., Nash S., Adams R. (2016). Derived neutrophil lymphocyte ratio is predictive of survival from intermittent therapy in advanced colorectal cancer: a post hoc analysis of the MRC COIN study. *British Journal of Cancer*.

[17] Terashima T., Yamashita T., Iida N. (2015). Blood neutrophil to lymphocyte ratio as a predictor in patients with advanced hepatocellular carcinoma treated with hepatic arterial infusion chemotherapy. *Hepatology Research*.

[18] Guthrie G. J. K., Charles K. A., Roxburgh C. S. D., Horgan P. G., McMillan D. C., Clarke S. J. (2013). The systemic inflammation-based neutrophil-lymphocyte ratio: experience in patients with cancer. *Critical Reviews in Oncology/Hematology*.

[19] Zhang X., Zhao X.-W., Liu D.-B. (2014). Lipid levels in serum and cancerous tissues of colorectal cancer patients. *World Journal of Gastroenterology*.

[20] van der Vorst E. P. C. (2020). High-density lipoproteins and apolipoprotein A1. *Sub-Cellular Biochemistry*.

[21] Duong P. T., Weibel G. L., Lund-Katz S., Rothblat G. H., Phillips M. C. (2008). Characterization and properties of pre*β*-HDL particles formed by ABCA1-mediated cellular lipid efflux to apoA-I. *Journal of Lipid Research*.

[22] Wang N., Silver D. L., Costet P., Tall A. R. (2000). Specific binding of ApoA-I, enhanced cholesterol efflux, and altered plasma membrane morphology in cells expressing ABC1. *The Journal of Biological Chemistry*.

[23] Liang H. Q., Rye K. A., Barter P. J. (1995). Cycling of apolipoprotein A-I between lipid-associated and lipid-free pools. *Biochimica et Biophysica Acta*.

[24] Sankaranarayanan S., Oram J. F., Asztalos B. F. (2009). Effects of acceptor composition and mechanism of ABCG1-mediated cellular free cholesterol efflux. *Journal of Lipid Research*.

[25] Hamilton K. K., Zhao J., Sims P. J. (1993). Interaction between apolipoproteins A-I and A-II and the membrane attack complex of complement. affinity of the apoproteins for polymeric C9. *The Journal of Biological Chemistry*.

[26] Tiniakou I., Drakos E., Sinatkas V. (2015). High-density lipoprotein attenuates Th1 and th17 autoimmune responses by modulating dendritic cell maturation and function. *Journal of Immunology*.

[27] Luo X.-L., Zhong G.-Z., Hu L.-Y. (2015). Serum apolipoprotein A-I is a novel prognostic indicator for non-metastatic nasopharyngeal carcinoma. *Oncotarget*.

[28] Borgquist S., Butt T., Almgren P. (2016). Apolipoproteins, lipids and risk of cancer. *International Journal of Cancer*.

[29] Bayerdörffer E., Mannes G. A., Richter W. O. (1993). Decreased high-density lipoprotein cholesterol and increased low-density cholesterol levels in patients with colorectal adenomas. *Annals of Internal Medicine*.

[30] Jung Y. S., Ryu S., Chang Y. (2015). Associations between parameters of glucose and lipid metabolism and risk of colorectal neoplasm. *Digestive Diseases and Sciences*.

[31] Gorphe P., Bouhir S., Garcia G. C. T. E. (2020). Anemia and neutrophil-to-lymphocyte ratio in laryngeal cancer treated with induction chemotherapy. *Laryngoscope*.

[32] Liu J., Li S., Zhang S. (2019). Systemic immune-inflammation index, neutrophil-to-lymphocyte ratio, platelet- to-lymphocyte ratio can predict clinical outcomes in patients with metastatic non-small-cell lung cancer treated with nivolumab. *Journal of Clinical Laboratory Analysis*.

[33] Cheong T. Y., Hong S. D., Jung K.-W., So Y. K. (2021). The diagnostic predictive value of neutrophil-to-lymphocyte ratio in thyroid cancer adjusted for tumor size. *PLoS One*.

[34] Verter E., Berger Y., Perl G. (2021). Neutrophil-to-lymphocyte ratio predicts recurrence pattern in patients with resectable colorectal liver metastases. *Annals of Surgical Oncology*.

