# Supplementary material for: The Prognostic Value of Serum Apolipoprotein A-I Level and Neutrophil-to-Lymphocyte Ratio in Colorectal Cancer Liver Metastasis
Source: J Oncol. 2022 Sep 27;2022:9149788. doi: 10.1155/2022/9149788 (PMC9532097; doi:10.1155/2022/9149788)
Supplement: Supplementary Materials — Normal values (ranges) of our laboratory. [file 9149788.f1.docx]

**Normal values (ranges) of our laboratory as follows:**

Apolipoprotein A-I 1.09-1.84（g/L）

high-density lipoprotein cholesterol ＞1.04（mmol/L）

neutrophil 2.0-7.5（E+9/L）

lymphocyte 1.6-4.0（E+9/L）
